# Soil microbial metabolism on carbon and nitrogen transformation links the crop-residue contribution to soil organic carbon

**DOI:** 10.1038/s41522-022-00277-0

**Published:** 2022-04-01

**Authors:** Zhihuang Xie, Zhenhua Yu, Yansheng Li, Guanghua Wang, Xiaobing Liu, Caixian Tang, Tengxiang Lian, Jonathan Adams, Junjie Liu, Judong Liu, Stephen J. Herbert, Jian Jin

**Affiliations:** 1 Key Laboratory of Mollisols Agroecology, Northeast Institute of Geography and Agroecology, Chinese Academy of Sciences, Harbin, 150081 China; 2 University of Chinese Academy of Sciences, Beijing, 100049 China; 3 Centre for AgriBioscience, La Trobe University, Bundoora, Vic 3086 Australia; 4 College of Agriculture, South China Agricultural University, Guangzhou, 510642 China; 5 School of Geographic and Oceanographic Sciences, Nanjing University, Nanjing, 210023 China; 6 Center for Agriculture, University of Massachusetts, Amherst, MA 01003 USA

**Keywords:** Soil microbiology, Microbial ecology, Microbiome

## Abstract

The beneficial effect of crop residue amendment on soil organic carbon (SOC) stock and stability depends on the functional response of soil microbial communities. Here we synchronized microbial metagenomic analysis, nuclear magnetic resonance and plant-^15^N labeling technologies to gain understanding of how microbial metabolic processes affect SOC accumulation in responses to differences in N supply from residues. Residue amendment brought increases in the assemblage of genes involved in C-degradation profiles from labile to recalcitrant C compounds as well as N mineralization. The N mineralization genes were correlated with the C and N accumulation in the particulate and mineral-associated C pools, and plant-derived aliphatic forms of SOC. Thus, the combined C and N metabolic potential of the microbial community transforms residue into persistent organic compounds, thereby increasing C and N sequestration in stable SOC pools. This study emphasizes potential microbially mediated mechanisms by which residue N affects C sequestration in soils.

## Introduction

With an annual production of approximately 5 × 10^9^ tons of crop residues in global farming systems, residue return into soil is a common practice to balance soil organic carbon (SOC) stock and maintain soil productivity^1–3^. Crop detritus has the potential to yield consistent suite of decomposition products and their sequestration in SOC pools^4–8^. This transformation is highly complex, being influenced by plant residue chemistry, soil microbiological processes and soil mineralogy^9,10^. The focus of research has gradually shifted from the humification processes of residue with respect to defining the continuum of SOC composition and its spatial distribution between particulate and mineral-associated pools, to the response of microbial communities to residue amendment^11–14^. This shift in focus has occurred because microbial community composition and functional activity have become widely recognized to be the fundamental mechanisms of residue-C transformation in soil^15,16^.

The N content of crop residues is considered as one of dominant factors affecting their degradability when these residues interact with soil microorganisms. An important observation has been that N-rich legume residues have faster degradation rates in soil compared to non-legume residues with high C/N ratio^17,18^. This suggests that residue-N may play an important role in soil C sequestration and SOC stability. Thus, an increasing assumption is that the potential stability of residue-induced C accumulated in soil would be attributed to the fact that residue-N and C correspondingly alter the SOC molecular composition and physico-spatial C distribution in SOC pools. This assumption has been made because organic molecules in soil differ in resilience to degradation by microorganisms. For example, plant-derived aliphatic compounds are more recalcitrant than O-alky compounds in soil^19^, and microbial accessibility to organic C varies between SOC pools^20^.

Debates on the importance of crop residue-N in the SOC sequestration and stability rest on a rather uncertain picture of microbial community composition and functional activity linking soil C and N dynamics. In spite of considerable progress on the research of residue-induced temporal shift of the phylogenetic composition of microbial communities in soils^11,21^, there is still lack of integrative work on soil microbial metagenomics to understand how C and N metabolisms link the residue-C flow into the SOC pools. With the aid of metagenomics, the entire gene family profile involved in the decomposition of a range of organic C compounds from labile (starch and cellulose) to recalcitrant sources (aromatics and lignin) and organic N mineralization (amino acids and protein) can be explored^22,23^, leaving this analytical potential to advance the knowledge of microbe-driven turnover of crop residue in farming soils.

Here, we argue that microbial metabolisms involved in residue C and N transformation are integrated to facilitate SOC sequestration and stability, which is greatly dependent on residue-N. In this study, ^15^N-labeled residues were amended into a severely degraded Mollisol due to intensive cultivation, which is the most typical fertile farming soil distributed across the world (accounting for 916 million ha globally)^24,25^. We used metagenomic analysis to characterize microbial functional gene profile and community composition, and subsequently assessed their contributions to residue-induced C accumulation in SOC pools and shifts in molecular composition of SOC. The resulting knowledge of the effects of soil microbial metabolisms on the C and N cycling may allow the development of mechanistic solutions to balancing SOC stock with crop residue return in agricultural systems.

## Results

### Total N and C concentrations in SOC fractions

**Fig. 1:**
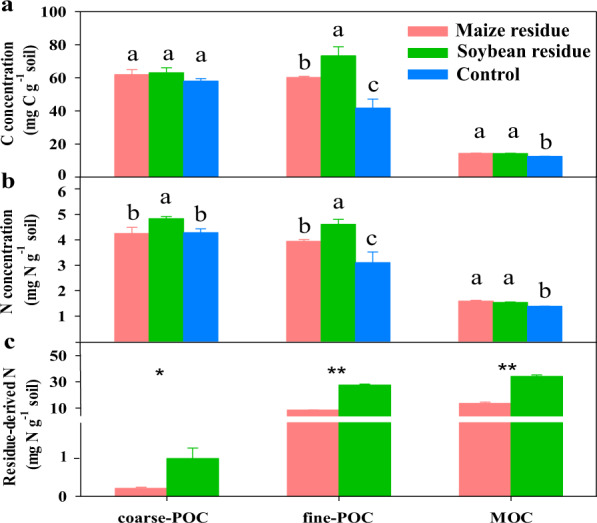
Carbon and nitrogen concentrations in various fractions of soil organic matter. The concentrations of carbon (**a**) and nitrogen (**b**) and residue-derived N (**c**) retained in the coarse particulate organic C (coarse-POC), fine POC (fine-POC) and mineral-associated organic C (MOC) fractions in soils amended without (control) and with maize and soybean residues after incubated at 25 °C for 250 days. Different letters above bars indicate a significant difference between treatments at *p* < 0.05 (one-way ANOVA). * and ** represent a significant difference analyzed by independent *t* test between treatments at *p* < 0.05 and *p* < 0.01, respectively. Error bars are the standard error of three replicates.

After 250 days of incubation, the residue amendment significantly increased the C concentration in the fine-POC and MOC fractions compared to the non-residue control. In particular, the C concentration in fine-POC increased by 41% under the soybean-residue treatment in comparison to 33% under the maize residue treatment (Fig. 1a).

A similar trend was observed for N concentration in those fractions in response to residue amendment with averaged 26.4% and 13.6% increases in fine-POC and MOC, respectively (Fig. 1b). In addition, residue amendment significantly decreased the available N concentration from 30 to 250 days of incubation compared to the control (Supplementary Fig. 1). Moreover, the available N concentration under the amendment of soybean residue was significantly higher than that under the amendment of maize residue (*p* < 0.05).

Residue-N was transformed and incorporated into various fractions. Soybean residue-N was mainly retained in the fine-POC and MOC fractions with 27.4 and 34.1 mg residue-N g^−1^, respectively, compared with 1.03 mg residue-N g^−1^ coarse-POC. The amount of soybean residue-N retained in each fraction was significantly higher than the amount of maize residue-N (Fig. 1c).

### Chemical composition of SOC

**Fig. 2:**
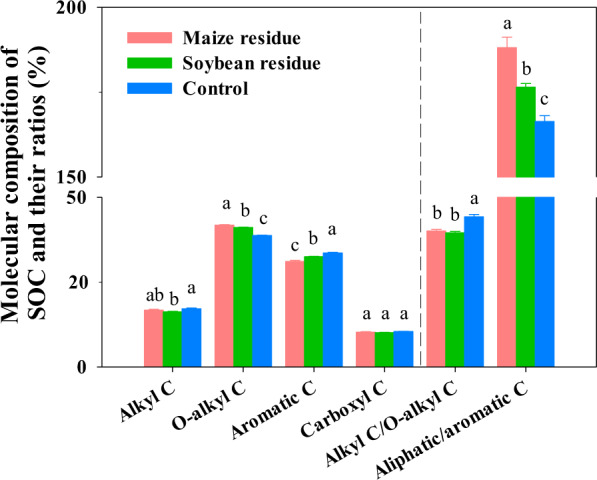
Chemical composition of soil organic C (SOC). The relative proportions (%) of different functional groups of SOC in response to the amendment of maize and soybean residues after incubation at 25 °C for 250 days. Different letters above bars indicate significant differences among treatments at *p* < 0.05 (one-way ANOVA). Error bars were the standard error of three replicates.

Residue amendment not only quantitively facilitated the C sequestration in soil, but also altered the quality of SOC. After 250 days of incubation, the residue amendment markedly altered the chemical compositions of SOC compared to the control (Fig. 2). As revealed by the NMR spectra, residue amendment significantly (*p* < 0.05) increased the relative proportion of O-alkyl C (labile C component) with 41.1 and 41.8% in the maize and soybean residue treatments, respectively, in comparison to 38.8% in the control. The aromatic C proportion (recalcitrant C component) in the maize and soybean residues treatments reached 31.1 and 32.5%, respectively, which were significantly lower than the control (33.6%). Residue amendment increased aliphatic/aromatic C ratio [aliphatic C (alkyl C+O-alkyl C)/aromatic C] and decreased the alkyl C/O-alkyl C ratio compared to the control.

### Microbial biomass C and activity

Residue amendment stimulated soil respiration, microbial biomass C and metabolic quotient over time compared to the control (Supplementary Fig. 2). The amendment of soybean residue resulted in a higher cumulative respiration than the maize residue amendment in the initial 137 days of incubation (*p* < 0.05), while an opposite trend was observed after 193 days of incubation (Supplementary Fig. 2a). Soil microbial biomass C on 7, 30 and 250 days, was significantly higher in the soybean residue treatment than in the maize residue treatment, but no difference was observed on 60 and 100 days (Supplementary Fig. 2b). In terms of the C use efficiency for microbial activity, the amendment of maize residue, however led to the greater metabolic quotient of soil microorganisms than the soybean residue treatment on 100 and 250 days (Supplementary Fig. 2c).

### Microbial community diversity

Residue amendment altered the alpha diversity of microbial community as reflected by the number of OTUs and Shannon index. A significant decrease of the OTU number was observed in response to residue amendment after 7 days of incubation, but no difference among the treatments by day 250. Both maize and soybean residue amendments significantly decreased Shannon index in the initial 30 days of incubation compared to the control, but this decrease only occurred in the maize residue treatment afterwards (Supplementary Fig. 3a). In the fungal community, the OTUs in the residue treatments were less than in the control until the end of incubation. The maize residue amendment significantly decreased the Shannon index at 30 days of incubation, but did not affect it thereafter compared to the control, while the soybean residue amendment lowered Shannon index (Supplementary Fig. 3b).

**Fig. 3:**
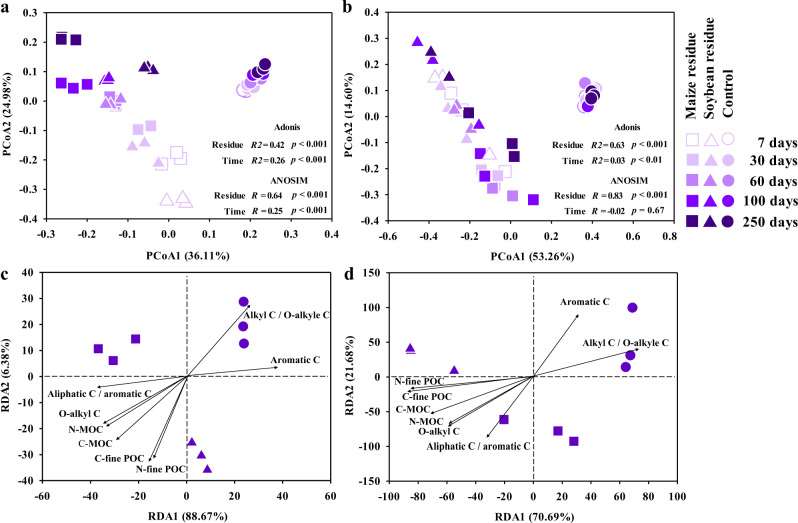
Microbial community composition. Principal coordinate analysis (PCoA) of bacterial (**a**) and fungal (**b**) communities in a Mollisol amended with maize and soybean residues for 7, 30, 60, 100 and 250 days (*n* = 3). Redundancy analysis (RDA) for the association of bacterial (**c**) and fungal (**d**) communities at the OTU level with C and N concentrations in the fine particulate (fine-POC) and mineral-associated C (MOC) fractions, and functional groups of SOC.

Regarding beta diversity, the PCoA analysis indicated that the clustering of bacterial community changed over time in the residue treatments (Fig. 3a). By 250 days of incubation, a significant difference in the community composition between the two residue treatments was found, as indicated by ANOSIM (R = 0.64, *p* < 0.001) and Adonis (R^2^ = 0.42, *p* < 0.001). By contrast, there was little change in the bacterial community of the control over time. Similarly, the fungal community composition was altered by the residue amendment and the significant difference between maize and soybean residue treatments occurred at the end of incubation (Fig. 3b).

The RDA revealed a strong association between the microbial community composition and the SOC composition and C concentration in SOC pools at 250 days of incubation (Fig. 3c and d). Specifically, the community composition under residue treatments had positive relationships with C and N concentrations in the fine POC and MOC fractions, and O-alkyl C proportion (*p* < 0.05), but had negative relationships with the aromatic C proportion and Alkyl C to O-alkyl C ratio (*p* < 0.05).

### Microbial metabolic profile

**Fig. 4:**
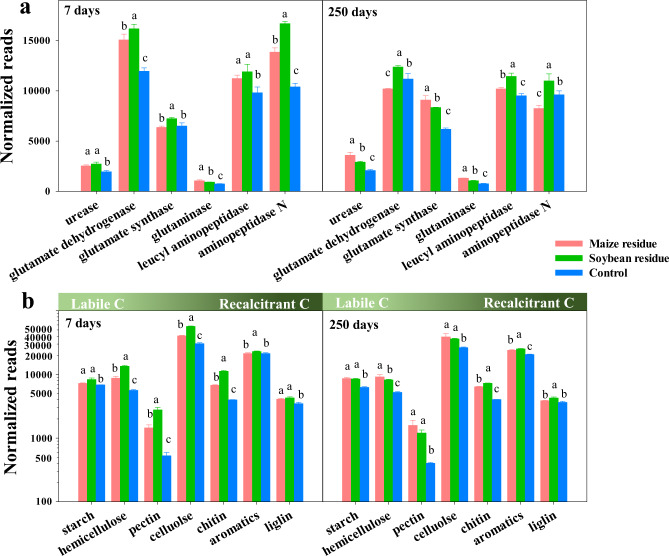
Microbial metabolic profile. Normalized reads of N-mineralization (**a**) and C-decomposition (**b**) genes in response to the amendment of maize and soybean residues after incubated at 25 °C for 7 and 250 days. Different letters above bars indicate significant differences among treatments at *p* < 0.05 (one-way ANOVA). Error bars are the standard error of three replicates.

The residue amendment increased the abundance of major N-ammonification genes at 7 and 250 days of incubation, with higher abundances of the functional genes coding urease, glutaminase and leucyl aminopeptidase compared to the control (Fig. 4a). The abundances of genes coding glutamate dehydrogenase, leucyl aminopeptidase and aminopeptidase were higher under the soybean than maize residue treatment at 250 days of incubation, while genes coding urease, glutamate synthase and glutaminase had an opposite trend.

Residue addition significantly increased the normalized read numbers of C-degradation genes compared to the control (Fig. 4b). On day 7 of incubation, the genes coding the mineralization of hemicellulose, pectin, cellulose and chitin had greater relative abundances under the soybean than maize residue treatment, while the similar abundances of the mineralization genes of chitin, aromatics and lignin were observed at day 250 (Supplementary Table 2).

### Microbial functional network associated with chemical composition of SOC and residue-N in SOC fractions

**Fig. 5:**
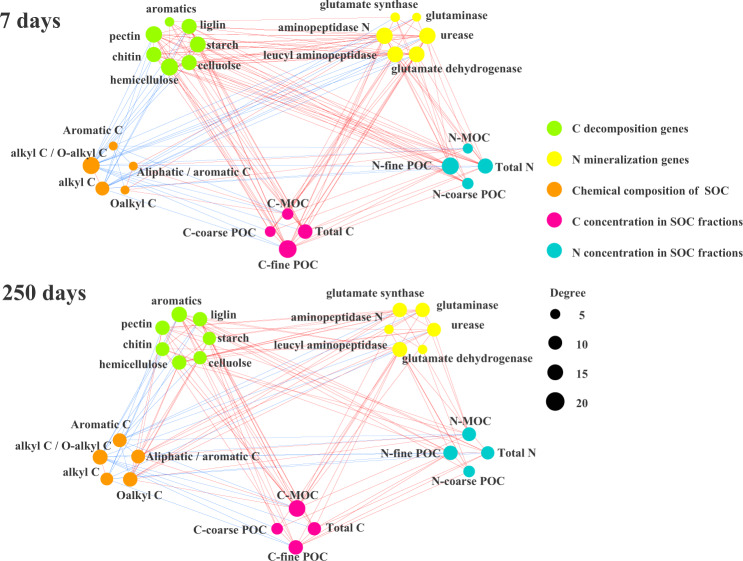
An overview of networks distributed by C-decomposition genes, N-mineralization genes, soil organic C (SOC) composition, C and N concentrations in SOC fractions after 7 and 250 days of incubation. The red lines indicate positive correlations between two individual nodes, whereas blue lines indicate negative correlations. The size of each node is proportional to the degree between two individual nodes. Total C, total N, C-coarse-POC, C-fine-POC, C-MOC, N-coarse-POC, N-fine-POC and N-MOC represent total C and N concentrations in soil, C and N concentrations in coarse-POC, fine-POC and MOC fractions, respectively.

The pattern of the network of microbial metabolic profile regarding the SOC composition and C and N concentrations in SOC fractions were similar between 7 and 250 days of incubation (Fig. 5 and Supplementary Table 3). This network demonstrated the close connection of C-decomposition and N-mineralization genes with soil C and N status. The SOC composition was also associated with C-decomposition genes. Specifically, based on the correlations of microbial metabolic profile with the composition of SOC and residue N in various SOC fractions, most of C-decomposition and N-mineralization genes were positively associated with the relative proportion of O-alkyl C but negatively with the alkyl C/O-alkyl C and the relative proportion of aromatic C (Supplementary Fig. 4a and b). The N-mineralization genes such as glutamate dehydrogenase, leucyl aminopeptidase and aminopeptidase N were positively associated with residue-N in fine-POC and MOC at 7 and 250 days of incubation (Supplementary Fig. 4c). Interestingly, the N concentration in the SOC fractions had more positive connections with C-decomposition genes compared to N-mineralization genes (Supplementary Table 3), given that most of C-decomposition genes were positively associated with N-mineralization genes (Supplementary Table 4).

### Keystone ecological clusters linking to C-decomposition and N-mineralization genes

**Fig. 6:**
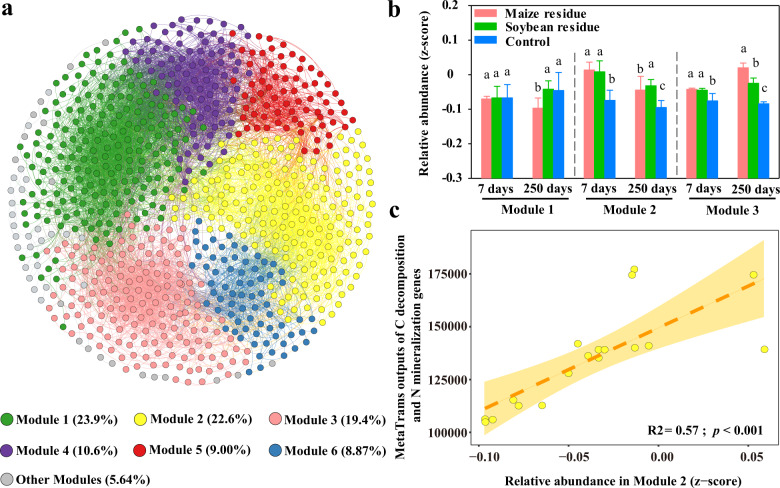
Ecological clusters based on multitrophic networks. Co-occurrence network diagram with nodes colored according to ecological clusters (**a**); the relative abundance of each module (**b**); the correlations of the relative abundance of Module 2 with normalized reads of C-decomposition and N-mineralization genes (**c**) in a Mollisol amended without (Control) and with maize and soybean residues for 7 and 250 days. Different letters above bars (**b**) indicate significant differences among treatments at *p* < 0.05 (one-way ANOVA). Error bars are the standard error of three replicates. The shading area (**c**) indicates 95% confidence interval.

Ecological networks were used to identify the functional potential of microbial clusters. The network of microbial community was separated into 6 major modules (Fig. 6a, Modularity = 0.60). The first 3 modules accounted for 65.9% of the network node numbers. Residue amendment significantly increased the relative abundance of modules 2 and 3 on days 7 and 250 of incubation (Fig. 6b). Among these modules, the relative abundance of module 2 was significantly correlated with normalized reads of C-decomposition and N-mineralization genes (Fig. 6c), indicating that module 2 is a keystone functional module. Moreover, the dominant bacterial genera in this module included *Massilia, Dyella, Luteimonas* and *Sphingomonas* affiliated to Proteobacteria, and *Granulicella* affiliated to Acidobacteria; the fungal genera included *Penicillium* and *Ramophialophora*. The details of C- and N-mineralization-associated genera within this module were shown in Supplementary Table 5, and most genera were significantly enriched under the treatment of residue amendment.

## Discussion

### Microbial community profiling relevant to residue-induced C sequestration

During the process of residue decomposition in soil, residue-N was pivotal to soil C sequestration in organic C pools. This is highlighted by the fact that N and C concentrations increased in SOC fractions in response to residue amendment. The application of N-enriched soybean residue led to a greater increase in the C concentration in POC compared to maize residue, and more soybean residue-N was observed in this fraction (Fig. 1), which further implied the fundamental contribution of residue-N to soil C accumulation in the POC pool.

Although differences in chemical properties of residues may influence the fate of residue-C in soil^26,27^, the C sequestration in SOC pools in response to residue amendment is mainly attributed to the nutrient stoichiometry within heterotrophic microorganisms^28,29^. Due to the greater C/N ratio of residues (50 to 120) than soil^11^, the transformation of residue C into the SOC pools is dominantly N-limited rather than C-limited^30^. This was indicated with the significant decrease of mineral N in the residue-amended soil compared to the control (Supplementary Fig. 1), suggesting that microorganisms may mine N from organic materials to meet their N requirement^31,32^.

Residue-induced differences in microbial community composition and metabolisms had important effects on the transformation of residue-C into SOC pools. The RDA in this study indicated the significant association between microbial community composition and C concentrations in POC and MOC (Fig. 3c and d). Our previous studies also found that bacterial community composition shifted considerably after 150 days of soybean residue amendment into a Mollisol^11,26^, which likely contributed to the accumulation of residue-C in SOC fractions^26^. Corresponding with the change in the microbial community composition, this study further demonstrated that residue amendments dramatically altered the community-level functional profile from labile to recalcitrant C mineralization (Fig. 4). Moreover, the range of C-mineralization genes were positively correlated with the C concentrations in SOC pools (Fig. 5 and Supplementary Table 3).

### The role of N-mineralization-associated metabolic profiles in C transformation

Microbial community function in relation to N mineralization was integrated with C transformation to contribute to the C accumulation in SOC pools in response to residue amendment. This is demonstrated by the fact that genes coding urease, glutamate synthase, glutaminase and aminopeptidase were significantly correlated with C-decomposition genes (Fig. 5 and Supplementary Table 4), C concentrations in POC and MOC fractions (Supplementary Fig. 5). Moreover, using GeoChip, He et al.^33^ demonstrated that significant relationships of microbial community functional structure with soil C and N dynamics. Taking this together, the increases in the normalized reads of aminopeptidase- and glutaminase-producing genes, and N concentration in SOC fractions in response to residue amendment, especially soybean residue amendment, suggest that microbial communities that metabolized residue-N were simultaneously acting with those on the residue C transformation. Moreover, a positive association of a number of N mineralization genes with microbial metabolic quotient (Supplementary Fig. 5) highlights the essential role of microbial N metabolisms in the reside-C utilized by microbial community. Therefore, residues may escalate the microbial community metabolic profile towards N mineralization, of which the magnitude of this stimulation on N mineralization genes may largely regulate the decomposition of the residue-C and subsequent sequestration in SOC pools.

The question is which genera were active in the C and N transformation in response to the residue amendment. In this study, a number of genera of key-stone phylotypes were positively associated with the abundance of C-decomposition and N-mineralization genes (Fig. 6 and Supplementary Table 5). *Massilia* and *Granulicella* are able to metabolize chitin for N and C sources^34–37^. *Dyella* and *Sphingomonas* as microbial resource can degrade a range of organic matters such as aromatic compounds^38^ and phenol^39^. *Luteimonas* played an important role in nitrate reduction in N cycle^40^. *Penicillium* was the predominant cellulolytic fungi that secrete enzymes to hydrolyze lignocellulose into usable sugars^41^. Those bacterial and fungal genera could be dominant functional producers manipulating the residue-C and N transformation.

### The role of microbial C and N metabolisms in SOC stability under residue amendment

The stabilization of residue-induced C sequestration in soil is relevant to the relative change of SOC composition, which was dominantly resulted from the microbial community function on the transformation of residue C and N. The proportional increase in aliphatic C group in response to reside amendment was considered to contribute to SOC stabilization as the aliphatic biomacromolecule is one of the major components in soil humic material that is a complex mixture of faunal, microbial and plant biopolymers and their degradation products^42–44^. The aliphatic biomacromolecules such as cutin, cutan and buerin are normally derived from plants and microorganisms, which may go through biochemical processes to form these compounds. These biochemical processes require microbial N metabolisms as well. The significant correlations of urease, glutamate synthase and glutaminase with aliphatic C and the aliphatic-to-aromatic C ratio in this study (Fig. 5 and Supplementary Fig. 4), clearly indicate the involvement of microbial N metabolisms in the contribution of residue-C transformation to SOC stability.

Moreover, the increase in MOC with residue amendment implies that those decomposed compounds may physically bind to soil minerals by ligand exchange, polyvalent cation bridges, van der Waals forces and H bonding^45–47^, which is resistant to enzymatic attack^48^. Under the residue amendment, the less increase in the abundance of genes involved in recalcitrant C degradation than labile C mineralization supports this view (Fig. 4 and Supplementary Table 2). A number of studies further reported that organic C bonded with clay and silt has longer turnover time than non-protected SOC^49,50^. In addition, more C occluded in the POC fraction with the amendment of N-enriched soybean residue than maize residue further demonstrated that residue-N-mediated C accumulation in this fraction may be isolated from decomposition due to aggregation^20,51,52^.

Collectively, the integrative work from microbial metagenomic analysis to SOC molecular composition advances our knowledge that crop residues accelerate microbial community metabolisms involved in the N mineralization, which is corresponding with labile and recalcitrant C transformation, and finally contribute to the C flow into SOC pools and the accumulation of persistent organic compounds in soil. Mechanistic understanding on the microbial N metabolism involvement in the crop residue turnover in soil would provide explanations for the benefits of N-enriched residues to the SOC stock and stability in farming soils.

## Methods

### Soil and residue preparation

The soil was collected from a farming paddock that was cultivated in a soybean-maize rotation without fertilization for six years (2012–2017) at Guangrong village in Hailun, Heilongjiang province in northeast China (47°21’N, 126°50’E). Soil type was a typical Mollisol or Phaeozem^53^. Soil samples were taken from the tillage layer (0–10 cm) at five random locations in an area of 120 m^2^ on the paddock before soybean was sown in 2018, and then pooled. In order to effectively remove gravels and organic debris and homogenize soil, the soil was partially air-dried to 32% of field water capacity and sieved through a 2-mm sieve. The partial drying (to 30% of field water capacity) has minimal impact on soil microbial properties^54^. The soil had a pH of 6.13, SOC of 23.3 mg g^−1^ and total N of 2.01 mg g^−1^.

The ^15^N-labeled crop residues were produced with soybean and maize plants grown in the Mollisol, in which Ca(^15^NO_3_)_2_ with 20% of ^15^N atom excess was homogeneously mixed at a rate of 100 mg N kg^−1^ soil before sowing. Six germinated seeds were sown in each pot (20 cm diameter and 40 cm high) containing 12 kg soil. The seedlings were thinned to 3 plants for soybean and 1 plant for maize after emergence. Three pots were set for each crop. Soil water content was maintained at 80 ± 5% of field capacity by weighing and watering. After plants matured, stalks were harvested, dried at 70 °C, ground through 2-mm and 0.25-mm sieves. The residues left between 0.25 and 2 mm were used for the following incubation experiment. The residue-C and N concentrations were 477 and 9.3 mg g^−1^ for the soybean residue (C/N = 51), and 431 and 4.1 mg g^-1^ for the maize residue (C/N = 105).

### Experimental design and sampling

An incubation experiment was carried out for 250 days with three treatments: (1) maize residue amendment, (2) soybean residue amendment and (3) no-residue control. There were 15 jars per treatment for five sampling dates. Thirty grams of air-dried soil was watered to 50% field water capacity and pre-incubated for 20 days at 25 °C to resume soil microbial activity^55^. Then 0.6 g of residue was mixed thoroughly with soil and put into a PVC core (5-cm height, 4-cm diameter) with a nylon mesh bottom. After the soil bulk density was adjusted to 1.1 g cm^−3^, the core was placed into a 1-L sealed Mason jar. A 50-mL vial containing 10 mL of 1 M NaOH to trap CO_2_ and a 20-mL vial containing 10 mL water were placed in each jar to maintain a high humidity. The incubation was conducted in the dark at 25 °C. Three blank controls without soil and residue were set up under the same condition. The soil water content was maintained at 60% of field water capacity through weighing and watering during the incubation.

At each of 7, 30, 60, 100 and 250 days of incubation, three jars/replicates from each treatment were randomly sampled. Soil from each jar was separated into three parts: one part (5 g) was air-dried for SOC fractionation and other chemical measurements including total C and N, functional groups of SOC and ^15^N abundance; one part (approximately 2 g) was stored at −80 °C for soil DNA extraction and Illumina sequencing; and the remaining part was freshly used for measurements of microbial biomass C (MBC) and available N.

### Measurements of soil respiration and biochemical properties

The NaOH vials were replaced every three days in the first two weeks, weekly from day 19 to day 117, every 10 days from day 118 to day 168, and every two weeks each time thereafter. Total CO_2_-C trapped in NaOH vials was determined using the titration method. Briefly, NaOH solution was precipitated using 1 mL of 1.0 M strontium chloride (SrCl_2_) solution, then titrated with 0.5 M HCl against a phenolphthalein indicator. The metabolic quotient (qCO_2_) was calculated as soil respiration divided by MBC^56^.

Soil pH was measured using a Wettler Toledo 320 pH meter in water extract (w:v = 1:5) after shaking the suspension for 30 min. The SOC and total soil N concentrations were measured with an Elementar III analyzer (Elementar Analysensysteme, Hanau, Germany). To avoid the interference of inorganic C in the measurement of SOC, the inorganic C in soil was removed using the HCI-fumigation method^57^. Briefly, soil samples were put into a microtiter plate and wetted to field capacity, then incubated in a sealed desiccator for 10 h with a beaker carrying the concentrated HCl solution (12 M). Thus, the soil carbonates were reacted to form CO_2_. Then, the soil samples were dried at 55 °C before measuring SOC. Soil available N was extracted from 2 g fresh soils in 20 mL of 0.5 M K_2_SO_4_ by shaking for 1 h, then measured using a flow-injection auto-analyser (SKALAR, San^++^, Netherlands). The atom% ^15^N in SOC fractions was determined using an isotope ratio mass spectrometer (Deltaplus, Finnigan MAT, Bremen, Germany).

The MBC was measured using the fumigation extraction method^58^. Total organic C in extracts was determined using an automated TOC analyzer (Shimadzu, TOC-VCPH, Japan). The MBC was calculated as the difference in TOC concentration in extracts between fumigated and non-fumigated soils divided by 0.38 (the extractable part of MBC after fumigation).

### SOC fractions

The SOC fractions at the end of the incubation were separated into coarse particulate organic C (>250 μm, coarse POC), fine POC (53–250 μm) and mineral-associated organic C (<53 μm, MOC)^59^. Briefly, 5 g soil was suspended in 40 mL of 1.7 g cm^−3^ sodium iodide (NaI) solution and centrifuged at 6500 × *g* for 1 h. The free light fraction (maize and soybean residues) was recovered from the solution surface. The heavy fraction was dispersed in 35 mL of 0.5 M sodium hexametaphosphate solution and shaken for 17 h (0.55 × *g*) on a reciprocal shaker (MX-RL-Pro DRAGON LAB, USA). The soil solution was passed through 250-μm and 53-μm sieves and then dried at 60 °C. The total C and N concentrations and atom% ^15^N in each fraction were measured using the methods described as above.

### Solid-state ^13^C NMR analysis

The chemical composition of SOC was determined by solid-state ^13^C nuclear magnetic resonance (NMR). Soil samples at the end of incubation were pre-treated with 2% hydrofluoric acid to remove paramagnetic materials^60^. Solid-state ^13^C NMR analyses were completed on a Bruker Avance Neo 400 NMR spectrometer (Billerica, USA). The samples were packed into 4-mm diameter zirconia rotors and spun at the rate of 8 kHz at the magic angle of 54.7°. Single contact times of 2 ms were applied, and a recycle delay of 2.5 s. Approximately 10,000 transients were collected to acquire spectra with a signal-to-noise ratio above 10.

After the baseline correction, the spectra were divided into four chemical shift regions, representing alkyl C (0–45 ppm), O-alkyl C (45–110 ppm), aromatic C (110–160 ppm), and carboxylic/amide/ester C (160–200 ppm)^61^. The ratios of alkyl C to O-alkyl C (alkyl C/O-alkyl C) and aliphatic C to aromatic C [aliphatic C (0–110 ppm)/aromatic C (110–160 ppm)], were used as indices to assess the degrees of stability and aliphaticity of soil^62^.

### DNA extraction and Illumina MiSeq Sequencing

Total soil DNA was extracted from 0.5 g fresh soil using a Fast DNA SPIN Kit for Soil (Qbiogene Inc., Carlsbad, CA, USA). Illumina MiSeq sequencing was performed by creating the amplicon libraries of bacterial 16 S rRNA gene with primers 515 (GTGCCAGCMGCCGCGG)/907R (CCGTCAATTCMTTTRAGTTT)^63^ and the internal transcribed spacer (ITS) region of fungi with primers ITS1F (CTTGGTCATTTAGAGGAAGTAA)/ITS2R (GCTGCGTTCTTCATCGATGC)^64^, respectively. The raw sequences of bacterial and fungi were uploaded onto the NCBI database under the accession number of PRJNA729887 and PRJNA729909, respectively.

The Quantitative Insights Into Microbial Ecology (QIIME) pipeline (http://qiime.org) was used for data analysis^65^. In brief, average quality score below 25 and reads shorter than 200 bp were removed. Among the remaining reads (24,250−44,588 for bacteria and 30,892−76,324 for fungi per sample), randomly selected subsets of 24,250 and 30,892 reads for bacteria and fungi were applied to each sample, and then clustered into operational taxonomic units (OTUs) on a 97% similarity level with UPARSE^66^. Each bacterial OTU was aligned them with the Greengenes 13_8 16 S rRNA database (http://greengenes.lbl.gov/)^67^. The UNITE database was used to determine fungal taxonomic identity^68^.

### Metagenome sequencing and bioinfomatimatic analyses

The DNA samples from days 7 and 250 were also used for microbial metagenomics analysis. We used the Covaris M220 (Gene Company Limited, China) to fragment DNA sample to an average size of about 400 bp for paired-end library construction. Libraries were sequenced using Illumina Hiseq Xten systems (Illumina Inc., San Diego, CA, USA) at Majorbio, Bio-Pharm Technology Co., Ltd. (Shanghai, China). Data were deposited in the NCBI Sequence Read Archive under the accession number PRJNA730191.

Clean reads were generated by removing adaptor sequences and low-quality reads from metagenome sequencing through fastp^69^ (https://github.com/OpenGene/fastp, version 0.20.0). MEGAHIT (parameters: kmer_min = 47, kmer_max = 97, step = 10) (https://github.com/voutcn/megahit, version 1.1.2) was used to assemble these high-quality reads to contigs, which makes use of succinct de Bruijn graphs. Then, we selected the contigs with the length of ≥300 bp as the final assembling result for gene prediction and functional annotation.

MetaGene^70^ (http://metagene.cb.k.u-tokyo.ac.jp/) was used to perform open reading frames (ORFs) of selected contigs. The predicted ORFs (length ≥ 100 bp) were selected and translated into amino acid sequences using the NCBI translation table (http://www.ncbi.nlm.nih.gov/Taxonomy/taxonomyhome.html/index.cgi?chapter=tgencodes#SG1).

We used CD-HIT^71^ (http://www.bioinformatics.org/cd-hit/, version 4.6.1) to construct a nonredundant gene catalog with 90% coverage and 90% sequence identity. Then, we mapped the reads to the constructed nonredundant gene catalog using SOAPaligner^72^ (http://soap.genomics.org.cn/, version 2.21) and calculated gene abundance. Functional annotation was conducted using Diamond^73^ (https://github.com/bbuchfink/diamond, version 0.8.35) against the Kyoto Encyclopedia of Genes and Genomes database (http://www.genome.jp/kegg/, version 94.2) with a cutoff e-value of 1e-5. We deployed the normalization of relative log expression (RLE) using the DESeq2 package (Version 1.14.1) on all genes^74^. The targeted KEGG Orthologies of marker genes involved in C and N metabolic pathways were selected as a subject database (Supplementary Table 1)^75–78^.

### Microbial community composition and network analyses

Principal coordinate analysis (PCoA) based on a UniFrac distance matrix and redundancy analysis (RDA) for bacterial and fungal communities was performed using the ‘vegan’^79^ and ‘GUniFrac’^80^ packages in R platform (version 3.2.5)^81^. The use of RDA in this study was to demonstrate the association of microbial community composition with soil chemical properties. Moreover, the analyses of similarities (ANOSIM) and Adonis were performed to statistically assess the difference in the microbial community composition between treatments using ‘vegan’ package^79^. The Pearson correlations of the read number of C- and N-mineralization genes with relative proportion of chemical functional group in SOC composition and C concentration in SOC pools were visualized on heatmaps.

Co-occurrence networks for bacterial and fungal communities were constructed using the R package “WGCNA”^82^. To reduce the complexity of the data sets, we removed OTUs with the relative abundance of less than 0.01% for all samples, leaving 821 bacterial and 144 fungal OTUs for further analyses. Pairwise Spearman correlations between nodes were obtained, and correlations with *r* > 0.8 and *p* < 0.05 were included in the network. Network modules were visualized using Gephi platform (version 0.9.2). Modules (ecological clusters) represent important ecological units with identifiable and highly connected taxa in each module^83,84^. The relative abundance of each module was computed using the standardized relative abundances (z-score) of the OTUs that belong to each module. Dominant functional genera that were associated with N mineralization and C decomposition were chosen from those ecological clusters with strong Spearman’s R^2^ > 0.7 at the significance of *p* < 0.05. In order to overcome the false positive problem in the multiple significance tests, the *p* values were adjusted to *q* values using False Discovery Rate (FDR) of 0.05 in the relevant analyses on C-decomposition genes and the C- and N-mineralization-associated genera.

### Calculations and data analysis

The δ^15^N (‰) in plant residues and SOC fractions was calculated according to the natural ^15^N abundance in the air^85^.

$$\delta ^{{{{\mathrm{15}}}}}{{{\mathrm{N = 1000}}}} \times \left( {{{{\mathrm{R}}}}_{{{{\mathrm{sample}}}}} - {{{\mathrm{R}}}}_{{{{\mathrm{standard}}}}}} \right){{{\mathrm{/R}}}}_{{{{\mathrm{standard}}}}}$$

where R_standard_ is the natural ^15^N abundance in the air (R_standard_ = 0.36647 atom%)^86^. R_sample_ is the ^15^N abundance in the SOC fractions.

The percentage of ^15^N-labeled residue-N (%Ndfr) in the SOC fractions was calculated as follows^87,88^:

$${{{\mathrm{\% Ndfr}}}} = \left( {\delta ^{{{{\mathrm{15}}}}}{{{\mathrm{N}}}}_{{{{\mathrm{sample}}}}} - \delta ^{{{{\mathrm{15}}}}}{{{\mathrm{N}}}}_{{{{\mathrm{soil}}}}}} \right){{{\mathrm{/}}}}\left( {\delta ^{{{{\mathrm{15}}}}}{{{\mathrm{N}}}}_{{{{\mathrm{residue}}}}} - \delta ^{{{{\mathrm{15}}}}}{{{\mathrm{N}}}}_{{{{\mathrm{soil}}}}}} \right)$$

where δ^15^N_sample_ is the δ^15^N value of SOC fractions with residue addition; δ^15^N_soil_ is the δ^15^N value without residue addition; and δ^15^N_residue_ is the δ^15^N value of the applied maize or soybean residue.

N residual proportion of ^15^N-labeled residue (RPN_r_) in SOC fractions:

$${{{\mathrm{RPN}}}}_{{{\mathrm{r}}}} = {{{\mathrm{100}}}} \times \left( {{{{\mathrm{N}}}}_{{{{\mathrm{soc}}}}} \times {{{\mathrm{\% Ndf}}}}_{{{\mathrm{r}}}}} \right){{{\mathrm{/N}}}}_{{{\mathrm{r}}}}$$

where N_soc_ is the total soil N content in SOC fractions; and N_r_ is the N content of ^15^N-labeled maize or soybean residue.

The amount of residue-N (in grams of residue-N per kilogram of soil) incorporated into the SOC fractions was calculated as follows:

$${{{\mathrm{Residue - N}}}} = {{{\mathrm{RPN}}}}_{{{\mathrm{r}}}} \times {{{\mathrm{N}}}}_{{{{\mathrm{soc}}}}}$$

where N_soc_ indicates the concentration of total N in each SOC fraction.

Data in this study were expressed as the mean ± standard error. Statistical significance was analyzed by one-way analysis of variance for three treatments and independent *t* test for two treatments after normal distribution test using SPSS software (version 19.0).

### Reporting summary

Further information on research design is available in the Nature Research Reporting Summary linked to this article.

## Supplementary information

Supplementary Materials

Reporting Summary

## Data Availability

The sequencing data of bacteria, fungi and metagenome are available in the NCBI database with the Sequence Read Archive (SRA) accession number of PRJNA729887, PRJNA729909 and PRJNA730191, respectively. The other datasets generated and/or analyzed in the current study are available from the corresponding author on reasonable request. R code is available at https://github.com/XIE-Zhihuang/npj-biofilms-and-microbiomes.

## References

[1] Cherubin MR (2018). Crop residue harvest for bioenergy production and its implications on soil functioning and plant growth: A review. Sci. Agric..

[2] Lal R (2005). World crop residues production and implications of its use as a biofuel. Environ. Int..

[3] Qu C, Li B, Wu H, Giesy JP (2012). Controlling air pollution from straw burning in China calls for efficient recycling. Environ. Sci. Technol..

[4] Baldock JA (1997). Assessing the extent of decomposition of natural organic materials using solid-state ^13^C NMR spectroscopy. Aust. J. Soil Res..

[5] Baldock JA, Masiello CA, Gelinas Y, Hedges JI (2004). Cycling and composition of organic matter in terrestrial and marine ecosystems. Mar. Cham..

[6] Fang Y (2019). Balancing nutrient stoichiometry facilitates the fate of wheat residue-carbon in physically defined soil organic matter fractions. Geoderma.

[7] Grandy AS, Neff JC (2008). Molecular C dynamics downstream: The biochemical decomposition sequence and its impact on soil organic matter structure and function. Sci. Total Environ..

[8] Mahieum N, Powlson DS, Randall EW (1999). Statistical analysis of published carbon-13 CPMAS NMR spectra of soil organic matter. Soil Sci. Soc. Am. J..

[9] Ahmad R, Nelson PN, Kookana RS (2006). The molecular composition of soil organic matter as determined by ^13^C NMR and elemental analyses and correlation with pesticide sorption. Eur. J. Soil Sci..

[10] Hall SJ, Ye CL, Weintraub SR, Hockaday WC (2020). Molecular trade-offs in soil organic carbon composition at continental scale. Nat. Geosci..

[11] Lian T (2017). The fate of soybean residue-carbon links to changes of bacterial community composition in Mollisols differing in soil organic carbon. Soil Biol. Biochem..

[12] Rasmussen C (2018). Beyond clay: towards an improved set of variables for predicting soil organic matter content. Biogeochemistry.

[13] Lehmann J, Kleber M (2015). The contentious nature of soil organic matter. Nature.

[14] Cotrufo MF, Ranalli MG, Haddix ML, Six J, Lugato E (2019). Soil carbon storage informed by particulate and mineral-associated organic matter. Nat. Geosci..

[15] Kong Y (2020). DNA stable-isotope probing delineates carbon flows from rice residues into soil microbial communities depending on fertilization. Appl. Environ. Microbiol..

[16] Zheng H (2021). Network analysis and subsequent culturing reveal keystone taxa involved in microbial litter decomposition dynamics. Soil Biol. Biochem..

[17] Blair N, Faulkner RD, Till AR, Sanchez P (2005). Decomposition of ^13^C and ^15^N labelled plant residue materials in two different soil types and its impact on soil carbon, nitrogen, aggregate stability, and aggregate formation. Aust. J. Soil Res..

[18] Ma Q, Watanabe T, Zheng J, Funakawa S (2021). Interactive effects of crop residue quality and nitrogen fertilization on soil organic carbon priming in agricultural soils. J. Soil Sediment..

[19] Lorenz K, Lal R, Preston CM, Nierop KGJ (2007). Strengthening the soil organic carbon pool by increasing contributions from recalcitrant aliphatic bio(macro)molecules. Geoderma.

[20] Six J, Bossuyt H, Degryze S, Denef K (2004). A history of research on the link between (micrco)aggregates, soil biota, and soil organic matter dynamics. Soil . Res..

[21] Lian T (2019). The shift of bacterial community composition magnifies over time in response to different sources of soybean residues. Appl. Soil Ecol..

[22] Zhou J (2012). Microbial mediation of carbon-cycle feedbacks to climate warming. Nat. Clim. Change.

[23] Ma Q (2020). Long-term farmyard manure application affects soil organic phosphorus cycling: A combined metagenomic and ^33^P/^14^C labelling study. Soil Biol. Biochem..

[24] Li M, He P, Guo X, Zhang X, Li L (2021). Fifteen-year no tillage of a Mollisol with residue retention indirectly affects topsoil bacterial community by altering soil properties. Soil Res.

[25] Liu X (2012). Overview of Mollisols in the world: Distribution, land use and management. Can. J. Soil Sci..

[26] Lian T (2016). Carbon input from ^13^C-labelled soybean residues in particulate organic carbon fractions in a Mollisol. Biol. Fertil. Soils.

[27] Wang Y (2017). Microbial association with the dynamics of particulate organic carbon in response to the amendment of elevated CO_2_-derived wheat residue into a Mollisol. Sci. Total Environ..

[28] Schneider T (2012). Who is who in litter decomposition? Metaproteomics reveals major microbial players and their biogeochemical functions. ISME J..

[29] Creamer CA (2015). Microbial community structure mediates response of soil C decomposition to litter addition and warming. Soil Biol. Biochem..

[30] Henriksen TM, Breland TA (1999). Evaluation of criteria for describing crop residue degradability in a model of carbon and nitrogen turnover in soil. Soil Biol. Biochem..

[31] Lu J, Dijkstra FA, Wang P, Cheng W (2019). Roots of non-woody perennials accelerated long-term soil organic matter decomposition through biological and physical mechanisms. Soil Biol. Biochem..

[32] Fontaine S (2011). Fungi mediate long term sequestration of carbon and nitrogen in soil through their priming effect. Soil Biol. Biochem..

[33] He Z (2010). Metagenomic analysis reveals a marked divergence in the structure of belowground microbial communities at elevated CO_2_. Ecol. Lett..

[34] Klarenberg IJ, Keuschnig C, Warshan D, Jonsdottir IS, Vilhelmsson O (2020). The total and active bacterial community of the chlorolichen *Cetraria islandica* and its response to long-term warming in sub-arctic tundra. Front. Microbiol..

[35] Faramarzi MA (2009). Optimization of cultural condtions for production of chitinase by a soil isolate of *Massilia timonae*. Biotechnol.

[36] Adrangi S, Faramarzi MA, Shahverdi AR, Sepehrizadeh Z (2010). Purification and characterization of two extracellular endochitinases from *Massilia timonae*. Carbohydr. Res..

[37] Tkacz A, Cheema J, Chandra G, Grant A, Poole PS (2015). Stability and succession of the rhizosphere microbiota depends upon plant type and soil composition. ISME J..

[38] Li A (2012). Metabolic characterization and genes for the conversion of biphenyl in Dyella ginsengisoli La-4. Biotechnol. Bioeng..

[39] Gong BN (2016). Enhanced degradation of phenol by *Sphingomonas* sp. GY2B with resistance towards suboptimal environment through adsorption on kaolinite. Chemosphere.

[40] Zhang D (2010). *Luteimonas terricola* sp. nov., a psychrophilic bacterium isolated from soil. Int. J. Syst. Evol. Microbiol.

[41] Zhang Z (2014). Predominance of *Trichoderma* and *Penicillium* in cellulolytic aerobic filamentous fungi from subtropical and tropical forests in China, and their use in finding highly efficient β-glucosidase. Biotechnol. Biofuels..

[42] Piccolo A, Spaccini R, Haberhauer G, Gerzabek MH (1999). Increased sequestration of organic carbon in soil by hydrophobic protection. Sci. Nat..

[43] Doerr SH (2005). Extraction of compounds associated with water repellency in sandy soils of different origin. Aust. J. Soil Res..

[44] Lorenz K, Lal R, Preston CM, Nierop K (2007). Strengthening the soil organic carbon pool by increasing contributions from recalcitrant aliphatic bio(macro)molecules. Geoderma.

[45] Jastrow, J. D. & Miller, R. M. Soil aggregate stabilization and carbon sequestration: feedbacks through organomineral associations. In: Lal R., Kimble J. M., Follett R. F., Stewart B. A., editors. Soil processes and the carbon cycle. Boca Raton, FL: CRC Press; p. 207–223 (1997).

[46] Dungait JAJ, Hopkins DW, Gregory AS, Whitmore AP (2012). Soil organic matter turnover is governed by accessibility not recalcitrance. Glob. Chang. Biol..

[47] Sun H (2019). Soil organic carbon stabilization mechanisms in a subtropical mangrove and salt marsh ecosystems. Sci. Total Environ..

[48] Ekschmitt K (2008). Soil-carbon preservation through habitat constraints and biological limitations on decomposer activity. J. Plant Nutr. Soil Sci..

[49] Eusterhues K, Rumpel C, Kleber M, Kögel‐Knabner I (2003). Stabilisation of soil organic matter by interactions with minerals as revealed by mineral dissolution and oxidative degradation. Org. Geochem..

[50] Wagai R, Mayer LM, Kitayama KN (2009). Nature of the “occluded” low-density fraction in soil organic matter studies: A critical review. Soil Sci. Plant Nutr..

[51] Gupta VVSR, Germida JJ (2015). Soil aggregation: Influence on microbial biomass and implications for biological processes. Soil Biol. Biochem..

[52] Six J, Conant RT, Paul EA, Paustian K (2002). Stabilization mechanisms of soil organic matter: implications for C-saturation of soils. Plant Soil.

[53] FAO–UNESCO. Soil Map of the World (1: 5000000), Vol. 1: Legend. UNESCO, Paris (1974).

[54] Meisner A, Leizeaga A, Rousk J, Baath E (2017). Partial drying accelerates bacterial growth recovery to rewetting. Soil Biol. Biochem..

[55] Butterly CR (2016). Long-term effects of elevated CO_2_ on carbon and nitrogen functional capacity of microbial communities in three contrasting soils. Soil Biol. Biochem..

[56] Wardle DA, Ghani A (1995). A critique of the microbial metabolic quotient (qCO_2_) as a bioindicator of disturbance and ecosystem development. Soil Biol. Biochem..

[57] Harris D, Horwáth WR, van Kessel C (2001). Acid fumigation of soils to remove carbonates prior to total organic carbon or carbon-13 isotopic analysis. Soil Sci. Soc. Am. J..

[58] Wu J, Joergensen RG, Pommerening B, Chaussod R, Brookes PC (1990). Measurement of soil microbial biomass C by fumigation-extration—an automated procedure. Soil Biol. Biochem..

[59] Cambardella CA, Elliott ET (1992). Particulate soil organic matter changes across a grassland cultivation sequence. Soil Sci. Soc. Am. J..

[60] Skjemstad JO, Clarke P, Taylor JA, Oades JM, Newman RH (1994). The removal of magnetic materials from surface soils. A solid state ^13^C CP/MAS NMR study. Aust. J. Soil Res.

[61] Chen C, Xu Z, Mathers N (2004). Soil carbon pools in adjacent natural and plantation forests of subtropical Australia. Soil Sci. Soc., Am., J..

[62] Fang Y (2020). Balanced nutrient stoichiometry of organic amendments enhances carbon priming in a poorly structured sodic subsoil. Soil Biol. Biochem..

[63] Biddle JF, Fitz-Gibbon S, Schuster SC, Brenchley JE, House CH (2008). Metagenomic signatures of the Peru Margin subseafloor biosphere show a genetically distinct environment. Proc. Natl Acad. Sci. USA.

[64] Ghannoum MA (2010). Characterization of the oral fungal microbiome (mycobiome) in healthy individuals. PLoS Pathog..

[65] Caporaso JG (2010). QIIME allows analysis of high-throughput community sequencing data. Nat. Methods.

[66] Edgar RC (2013). UPARSE: highly accurate OTU sequences from microbial amplicon reads. Nat. Methods.

[67] McDonald D (2011). An improved Greengenes taxonomy with explicit ranks for ecological and evolutionary analyses of bacteria and archaea. ISME J..

[68] Kõljalg U (2005). UNITE: a database providing web-based methods for the molecular identification of ectomycorrhizal fungi. New Phytol..

[69] Chen S, Zhou Y, Chen Y, Gu J (2018). fastp: an ultra-fast all-in-one FASTQ preprocessor. Bioinformatics.

[70] Noguchi H, Park J, Takagi T (2006). MetaGene: prokaryotic gene finding from environmental genome shotgun sequences. Nucleic Acids Res..

[71] Fu L, Niu B, Zhu Z, Wu S, Li W (2012). CD-HIT: accelerated for clustering the next-generation sequencing data. Bioinformatics.

[72] Li R, Li Y, Kristiansen K, Wang J (2008). SOAP: short oligonucleotide alignment program. Bioinformatics.

[73] Buchfink B, Xie C, Huson DH (2015). Fast and sensitive protein alignment using DIAMOND. Nat. Methods.

[74] Love MI, Huber W, Anders S (2014). Moderated estimation of fold change and dispersion for RNA-seq data with DESeq2. Genome Biol..

[75] Nelson MB, Martiny AC, Martiny JBH (2016). Global biogeography of microbial nitrogen-cycling traits in soil. Proc. Natl. Acad. Sci. USA.

[76] Nelson MB, Berlemont R, Martiny AC, Martiny JBH (2015). Nitrogen cycling potential of a grassland litter microbial community. Appl. Environ. Microbiol..

[77] Xue K (2016). Tundra soil carbon is vulnerable to rapid microbial decomposition under climate warming. Nat. Clim. Change.

[78] Yuan M (2018). Microbial functional diversity covaries with permafrost thaw-induced environmental heterogeneity in tundra soil. Glob. Change Biol..

[79] Oksanen, J. et al. Vegan: Community ecology package. R package version 2.5-3. (2018). https://CRAN.R-project.org/package=vegan.

[80] Oksanen, J. GUniFrac: Generalized UniFrac distances. R package version 1.0. (2012). https://cran.r-project.org/web/packages/GUniFrac/index.html.

[81] R Development Core Team. Vienna, Austria: R Foundation for Statistical Computing. (2016).

[82] Langfelder P, Horvath S (2012). Fast R functions for robust correlations and hierarchical clustering. J. Stat. Softw..

[83] Heleno R, Devoto M, Pocock M (2012). Connectance of species interaction networks and conservation value: Is it any good to be well connected?. Ecol. Indic..

[84] Menezes AB (2015). Network analysis reveals that bacteria and fungi form modules that correlate independently with soil parameters. Environ. Microbiol..

[85] Robinson D (2001). δ^15^N as an integrator of the nitrogen cycle. Trends Ecol. Evol..

[86] Werner RA, Brand WA (2001). Referencing strategies and techniques in stable isotope ratio analysis. Rapid Commun. Mass Spectrom..

[87] Wang Y (2019). Dynamics of maize straw-derived nitrogen in soil aggregates as affected by fertilization. J. Soil Sediment..

[88] Ding W, Li S, He P, Huang S (2019). Contribution and fate of maize residue-^15^N and urea-^15^N as affected by N fertilization regime. PLoS One.

