# Supplementary material for: Soil microbial metabolism on carbon and nitrogen transformation links the crop-residue contribution to soil organic carbon
Source: NPJ Biofilms Microbiomes. 2022 Apr 1;8:14. doi: 10.1038/s41522-022-00277-0 (PMC8975862; doi:10.1038/s41522-022-00277-0)
Supplement: Supplementary file 1 — Supplementary Materials [file 41522_2022_277_MOESM1_ESM.pdf]

Soil microbial metabolism on carbon and nitrogen transformation links the crop-residue contribution to soil organic carbon

Zhihuang Xie†, Zhenhua Yu†, Yansheng Li, Guanghua Wang, Xiaobing Liu, Caixian Tang, Tengxiang Lian, Jonathan Adams, Junjie Liu, Judong Liu, Stephen J Herbert, Jian Jin *

†Xie Z and Yu Z contribute to this work equally.
*Corresponding author. Email: jinjian29@hotmail.com

This PDF file includes:

Figures S1 to S5
Tables S1 to S5

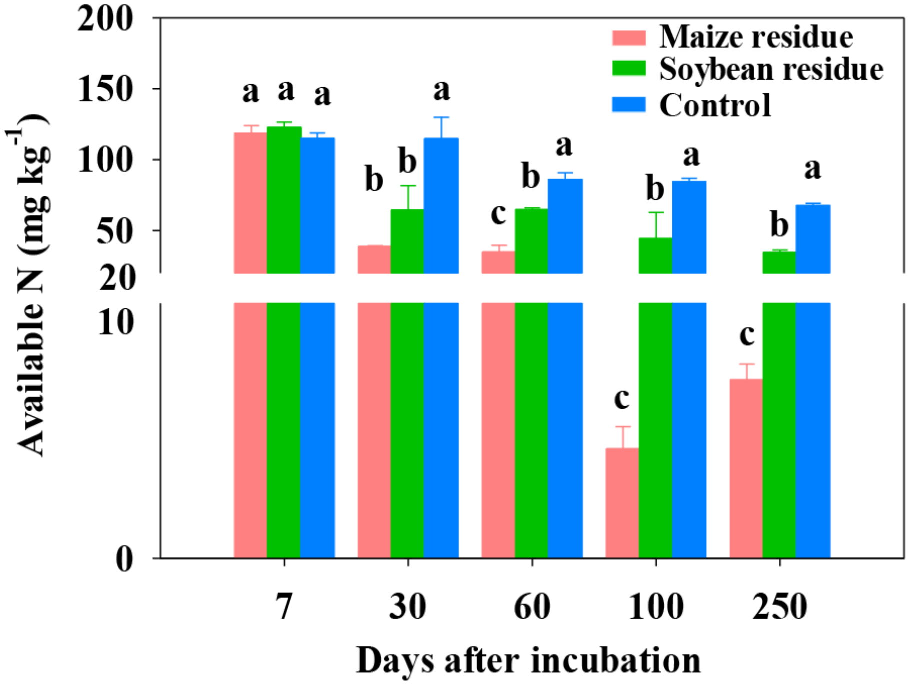

Supplementary Fig. 1 Soil available N. Available N concentration in a Mollisol amended without (control) and with maize and soybean residues after incubated at 25°C for 250 days. Different letters above bars indicate a significant difference between treatments at $p < 0.05$ (one-way ANOVA). Error bars are the standard error of three replicates.

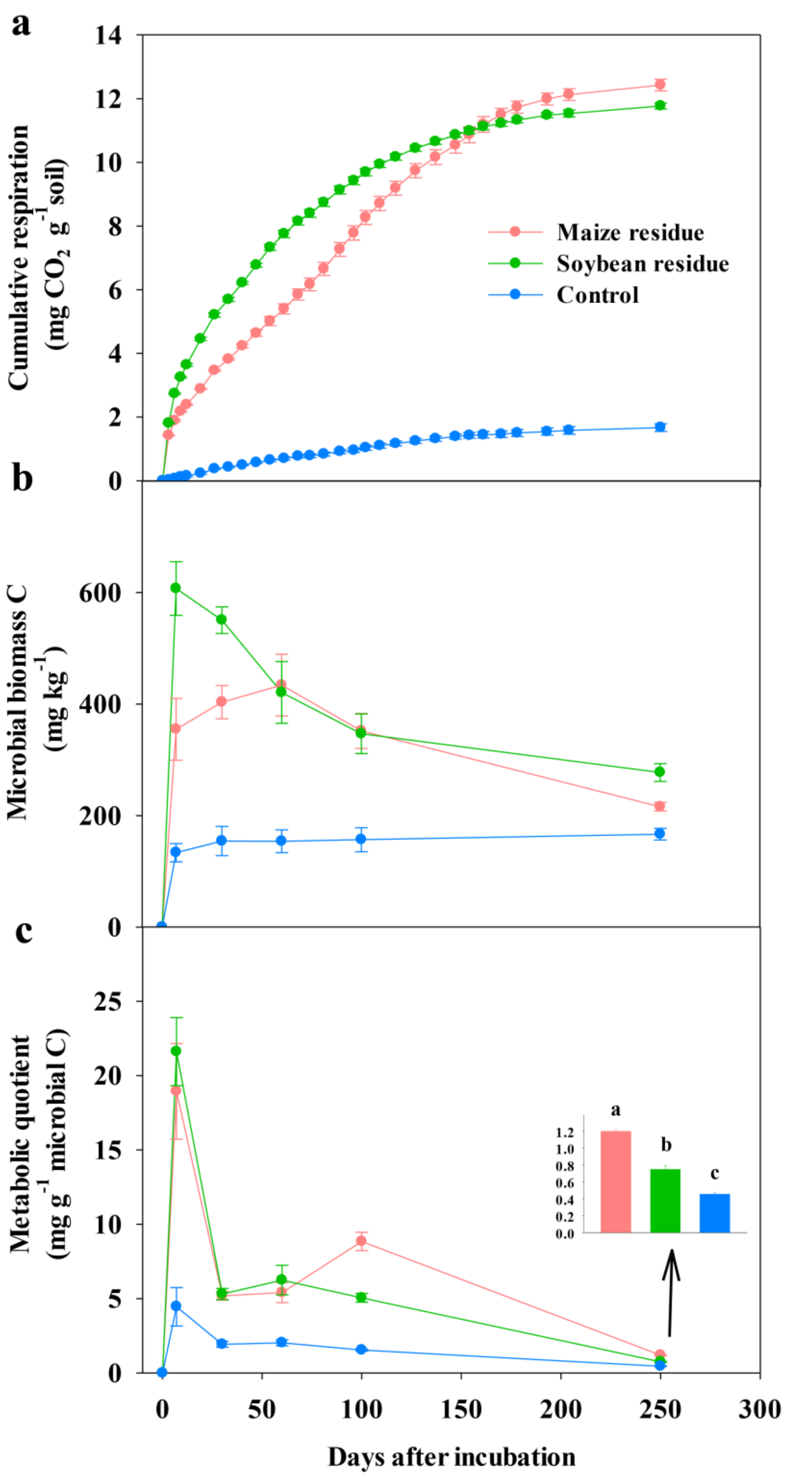

Supplementary Fig. 2 Soil cumulative respiration, microbial biomass C and metabolic quotient. The cumulative respiration (**a**), microbial biomass C (**b**) and metabolic quotient (**c**) in soils amended without (control) and with maize and soybean residues after incubated at 25°C for 250 days. Different letters above bars indicate a significant difference between treatments at $p < 0.05$ (one-way ANOVA). Error bars are the standard error of three replicates.

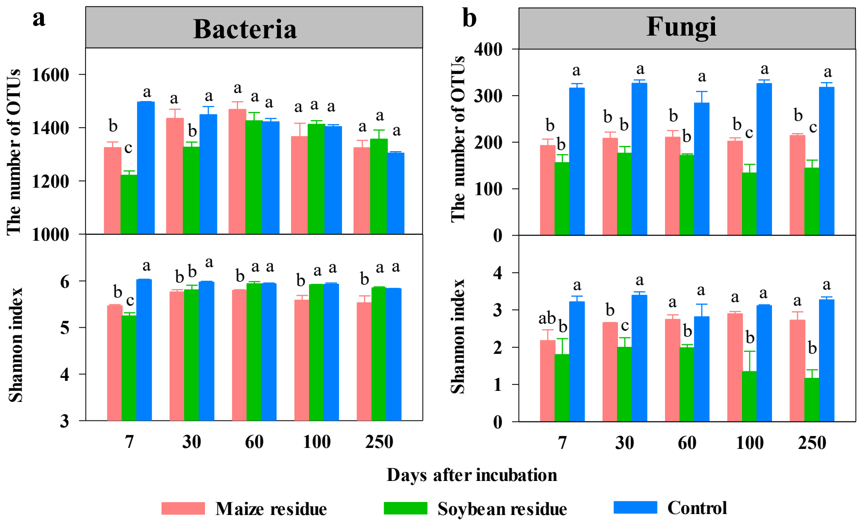

Supplementary Fig. 3 Alpha diversity indices. The number of OTUs and Shannon index of bacterial (**a**) and fungal (**b**) communities in a Mollisol amended without (Control) and with maize and soybean residues over 250 days. Different letters above bars indicate a significant difference between treatments at $p < 0.05$ (one-way ANOVA). Error bars are the standard error of three replicates.

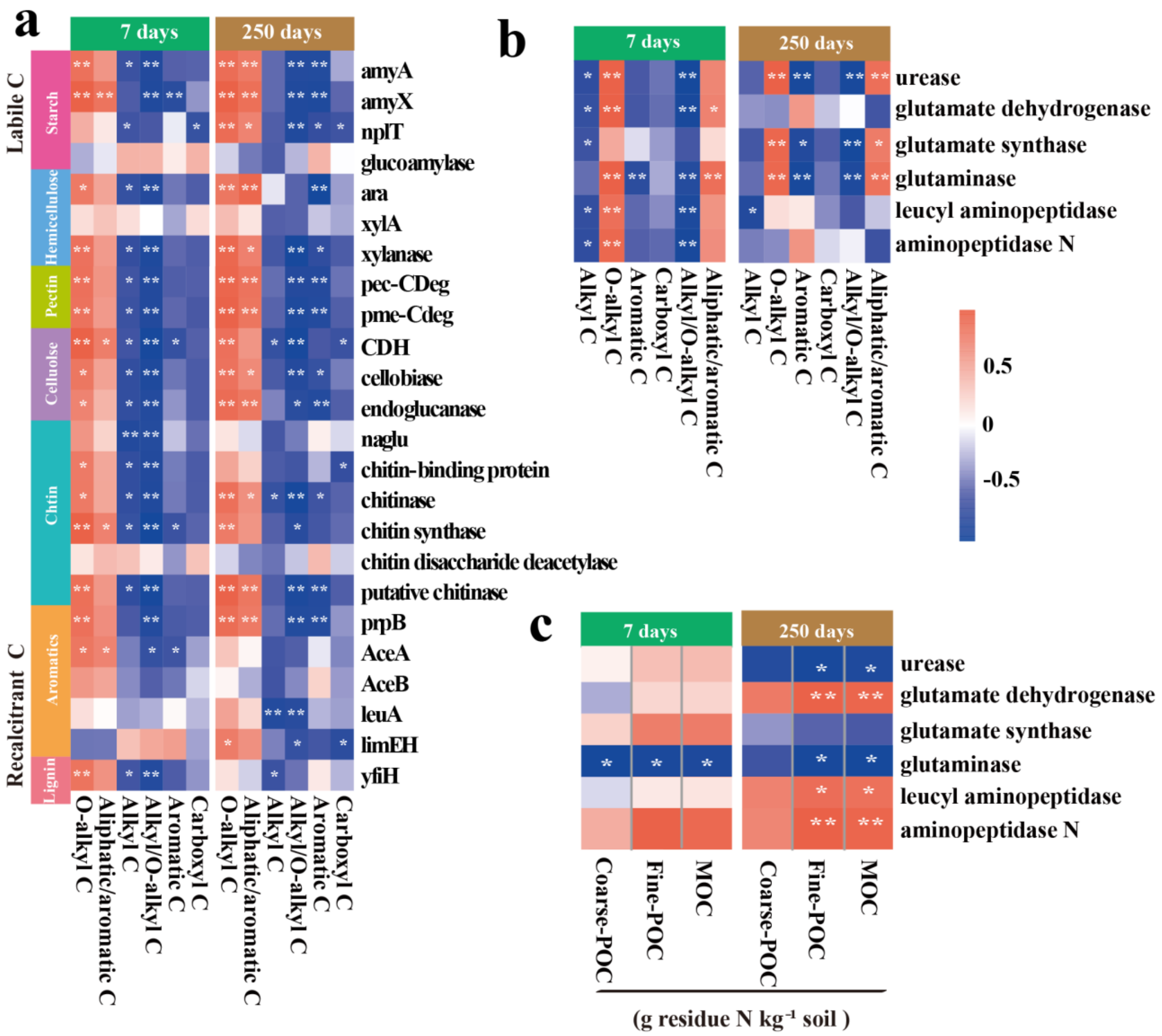

Supplementary Fig. 4 Pearson correlations of microbial metabolic profile with the composition of soil organic C (SOC) and residue-N in SOC pools. Pearson correlations of the read number of C-decomposition (**a**) and N-mineralization genes with relative abundances of functional groups of SOC (**b**), and residue-derived N retained in coarse particulate organic (coarse-POC), fine POC (fine-POC) and mineral-associated organic C (MOC) fractions (**c**) in a Mollisol amended with maize and soybean residues after incubated at 25°C for 7 and 250 days. The color intensity in each cell indicates the *r* value. Blue color indicates negative correlations and red indicates positive correlations (from -1 to 1). * and ** represent significance at $p < 0.05$ and $p < 0.01$, respectively.

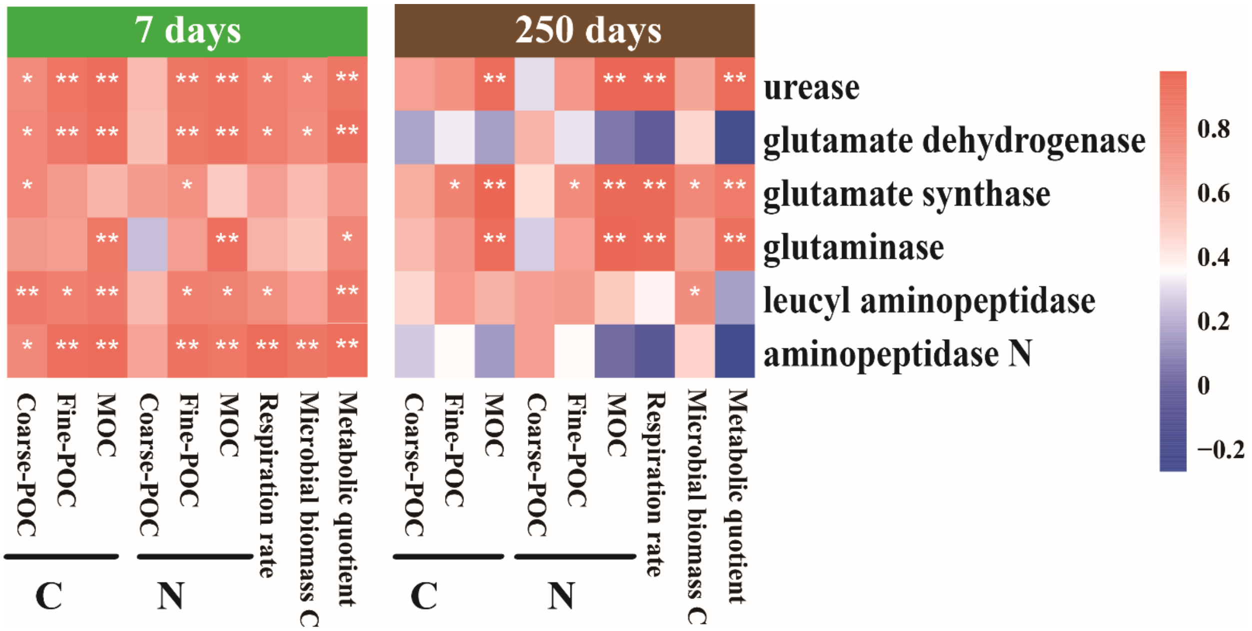

Supplementary Fig. 5 Pearson correlations of microbial metabolic profile with C and N concentrations in various fractions of soil organic C, and microbial properties. Pearson correlations of the read number of N-mineralization genes with C and N concentrations in coarse particulate organic C (coarse-POC), fine particulate organic C (fine-POC) and mineral-associated organic C (MOC) fractions, microbial respiration rate, biomass C and metabolic quotient in a Mollisol amended with maize and soybean residues after incubated at 25°C for 7 days and 250 days. The color intensity in each cell indicates the r value. Blue color indicates negative correlations, and red indicates positive correlations (from -1 to 1). *and ** and represent significant at levels $p < 0.05$ and $p < 0.01$, respectively.

Supplementary Table 1 N-mineralization and C-decomposition genes searched in the pathways and KEGG identification.

Gene category	Subcategory	Genes	KEGG identification
**N-mineralization**	Urease	urease subunit gamma/beta	K14048, K01430, K01429
		urease	K01427
		urease subunit alpha	K01428
	Glutamate dehydrogenase	glutamate dehydrogenase	K00260, K00261, K00262, K00371
	Glutamate synthase	glutamate synthase (NADPH/NADH) large chain	K00265
		glutamate synthase (NADPH/NADH)	K00264
		glutamate synthase (NADPH/NADH) small chain	K00266
		glutamate synthase (ferredoxin)	K00284
	Glutaminase	glutaminase	K01425
		glutamin-(asparagin-)ase	K05597
	Leucyl aminopeptidase	leucyl aminopeptidase	K01255
	Aminopeptidase N	aminopeptidase N	K01256
**C-decomposition**	Starch	amyA	K01176, K07405
		amyX	K01200
		nplT	K01208, K12575
		glucoamylase	K01178
	Cellulose	CDH	K19069
		cellobiase	K01188, K05349, K05350
		endoglucanase	K01179
	Hemicellulose	*ara*	K20844
		xylA	K01805
		xylanase	K01181
	Chitin	naglu	K01205
		chitin-binding protein	K03933
		chitinase	K01183
		chitin synthase	K00698
		chitin disaccharide deacetylase	K03478
		putative chitinase	K03791
	Pectin	*pec*-CDeg	K01728
		pme-Cdeg	K01051
	Aromatics	prpB	K03417
		AceA	K01637
		AceB	K01638
		leuA	K01649
		limEH	K10533
	Lignin	yfiH	K05810

Supplementary Table 2 MetaTrans output of C-decomposition genes in response to the amendment of maize and soybean residues after incubated at 25°C for 7 and 250 days. Different letters indicate significant differences among treatments on each sampling day at $q < 0.05$ (one-way ANOVA). The q values are the adjusted p values using False Discovery Rate (FDR) of 0.05.

Subcategory	Genes	7 days				250 days			
		Maize residue	Soybean residue	Control	q values	Maize residue	Soybean residue	Control	q values
Starch	amyA	2379b	3092a	1688c	<0.001	2228a	1707b	1255c	<0.001
	amyX	104a	75.1a	7.13b	0.018	344a	178b	21.6c	0.002
	nplT	1273b	1905a	1246b	0.011	2238a	2156a	1098b	<0.001
	glucoamylase	3488b	3248c	3921a	0.002	3832b	4437a	3882b	0.017
Cellulose	CDH	124b	244a	0.65c	<0.001	53.8b	162a	0.63c	<0.001
	cellobiase	31868b	42017a	24789c	<0.001	29823a	28023a	21528b	0.006
	endoglucanase	8379b	14585a	5657c	<0.001	9192a	7909a	4789b	0.011
Hemicellulose	*ara*	1087b	2755a	356c	<0.001	1448a	510b	326b	0.002
	xylA	3433bc	3214b	3506a	0.060	3883a	4060a	3408b	0.034
	xylanase	4254b	7470a	1770c	<0.001	3815a	3643a	1490b	<0.001
Chitin	naglu	468b	849a	364b	<0.001	492ab	621a	459b	0.092
	chitin-binding protein	468b	1359a	138c	<0.001	242a	258a	163a	0.170
	chitinase	3171b	5661a	1762c	<0.001	3409a	3401a	1903b	<0.001
	chitin synthase	487b	719a	12.2c	<0.001	180b	568a	14.1c	<0.001
	chitin disaccharide deacetylase	1365a	1297a	1401a	0.320	1170b	1619a	1268b	0.007
	putative chitinase	788b	1331a	311c	<0.001	929a	794a	225b	<0.001
Pectin	*pec*-CDeg	667b	1400a	173c	<0.001	670a	421a	107b	0.007
	pme-Cdeg	784b	1370a	354c	<0.001	915a	784a	295b	<0.001
Aromatics	prpB	1021b	1203a	744c	0.002	1495a	1208b	794c	<0.001
	AceA	3644a	3555a	3269b	0.001	3785a	3884a	3381b	0.002
	AceB	7044a	7117a	6784a	0.260	6680b	7416a	6421c	<0.001
	leuA	9572b	10956a	10429a	0.017	11891a	12526a	9887b	0.0015
	limEH	41.4a	60.5a	64.5a	0.23	177a	199a	67.5b	0.042
Lignin	yfiH	4092a	4278a	3483b	0.0032	3865b	4271a	3636b	0.0039

Supplementary Table 3 Mantel test of microbial metabolic profile with C and N in various fractions and composition of soil organic C (SOC). Coefficients (r) and significant levels (*p*) of correlations, using the Mantel test of C-decomposition and N-mineralization genes with C and N retained in SOC fractions and chemical composition. Data with blue and yellow backgrounds were for 7 and 250 days of incubation at 25°C, respectively.

	**C decomposition genes**		**N mineralization genes**		**SOC composition**		**C in SOC fractions**		**N in SOC fractions**	
	r	*p*	*r*	*p*	*r*	*p*	*r*	*p*	*r*	*p*
C decomposition genes	-		**0.864**	**0.002**	**0.856**	**0.003**	**0.911**	**0.002**	**0.789**	**0.001**
N mineralization genes	**0.794**	**0.001**	-		**0.811**	**0.002**	**0.798**	**0.004**	**0.673**	**0.003**
SOC composition	**0.770**	**0.008**	**0.679**	**0.003**	-		**0.637**	**0.009**	**0.461**	**0.018**
C in SOC fractions	**0.772**	**0.005**	**0.477**	**0.011**	**0.637**	**0.009**	-		**0.874**	**0.001**
N in SOC fractions	**0.626**	**0.003**	**0.376**	**0.036**	**0.461**	**0.018**	**0.874**	**0.001**	-	

Significant correlations ($p < 0.05$) are shown in bold.

Supplementary Table 4 Pearson correlations (*r*) of total N-mineralization genes with C-decomposition genes in a Mollisol amended with maize and soybean residues after incubated at 25°C for 7 and 250 days

C-decomposition genes	7 days		250 days	
	r	*p*	*r*	*p*
Starch	**0.891**	**0.001**	**0.690**	**0.040**
Celluolse	**0.922**	**<0.001**	0.497	0.174
Hemicellulose	**0.927**	**<0.001**	0.479	0.192
Chitin	**0.913**	**0.001**	**0.767**	**0.016**
Pectin	**0.894**	**0.001**	0.371	0.325
Aromatics	**0.770**	**0.015**	**0.927**	**<0.001**
Liglin	**0.941**	**<0.001**	**0.944**	**<0.001**
Total	**0.929**	**<0.001**	0.662	0.052

Significant correlations ($p < 0.05$) are shown in bold.

Supplementary Table 5 The C- and N-mineralization-associated genera in Module 2 in a Mollisol amended with the maize and soybean residues and incubated at 25°C for 7 and 250 days. Significant levels of main effects of residue treatment and incubation time and their interactions are indicated. The *q* values are the adjusted *p* values using False Discovery Rate (FDR) of 0.05.

			7 days			250 days			two-way ANOVA (*q* values)		
	Phylum	Genus	Maize	Soybean	Control	Maize	Soybean	Control	Treatment	Time	Treatment × Time
Bacteria	Proteobacteria	*Massilia*	1.98	8.64	0.47	0.09	0.13	0.03	<0.001	<0.001	<0.001
		Dyella	2.01	5.17	0.52	0.25	0.27	0.35	<0.001	<0.001	<0.001
		Luteimonas	0.87	0.70	0.61	0.07	0.33	0.32	0.620	<0.001	0.007
		norank_f__Burkholderiaceae	0.38	0.93	0.09	0.09	0.03	0.02	0.003	<0.001	0.003
		unclassified_f__Burkholderiaceae	0.38	0.70	0.05	0.00	0.01	0.01	0.011	<0.001	0.012
		Sphingomonas	0.40	0.39	0.09	0.02	0.08	0.10	0.007	<0.001	<0.001
		Alsobacter	0.25	0.11	0.08	0.04	0.05	0.04	<0.001	<0.001	<0.001
		Lysobacter	0.10	0.11	0.03	0.00	0.01	0.00	0.005	<0.001	0.009
		norank_f__0319-6G20	0.11	0.10	0.02	0.00	0.01	0.00	0.017	<0.001	0.029
		norank_f__Caulobacteraceae	0.03	0.07	0.02	0.01	0.01	0.01	0.005	<0.001	0.011
		OM27_clade	0.04	0.02	0.02	0.00	0.01	0.00	0.330	<0.001	0.170
		Rubellimicrobium	0.03	0.02	0.01	0.00	0.01	0.01	0.260	<0.001	0.006
	Acidobacteria	*Granulicella*	0.33	0.67	0.30	0.02	0.15	0.18	0.015	<0.001	0.066
		Terriglobus	0.17	0.21	0.03	0.00	0.02	0.02	<0.001	<0.001	0.031
		norank _o__Acidobacteriales	1.63	1.53	1.22	0.74	0.54	0.61	0.044	<0.001	0.130
	Planctomycetes	*Singulisphaera*	0.06	0.04	0.03	0.01	0.01	0.01	<0.001	<0.001	<0.001
		norank_f__WD2101_soil_group	0.85	1.30	0.89	0.23	0.34	0.13	<0.001	<0.001	<0.001
	Bacteroidetes	*Mucilaginibacter*	0.03	0.06	0.01	0.00	0.00	0.00	<0.001	<0.001	0.003
		norank_f__Chitinophagaceae	0.04	0.09	0.01	0.00	0.00	0.00	0.006	<0.001	0.005
		norank _o__OPB56	0.03	0.03	0.02	0.00	0.00	0.00	0.560	<0.001	0.250
	Armatimonadetes	*norank _p__Armatimonadetes*	0.06	0.07	0.03	0.00	0.01	0.01	0.053	<0.001	0.056
	Actinobacteria	*Catenulispora*	0.04	0.09	0.02	0.00	0.01	0.01	0.004	<0.001	0.005
Fungi	Ascomycota	*Penicillium*	1.21	0.58	0.26	0.34	0.10	0.10	0.007	0.001	0.097
	Ascomycota	*Chaetomium*	0.10	0.25	0.01	0.00	0.01	0.02	0.020	0.002	0.014
	Ascomycota	*Ramophialophora*	0.58	1.60	0.04	0.03	0.01	0.02	0.490	0.180	0.440
