# Supplementary material for: Soil microbial metabolism on carbon and nitrogen transformation links the crop-residue contribution to soil organic carbon
Source: NPJ Biofilms Microbiomes. 2022 Apr 1;8:14. doi: 10.1038/s41522-022-00277-0 (PMC8975862; doi:10.1038/s41522-022-00277-0)
Supplement: Supplementary file 2 — Reporting Summary [file 41522_2022_277_MOESM2_ESM.pdf]

nature portfolio

Corresponding author(s): Jian Jin

Last updated by author(s): Oct 31, 2021

Reporting Summary

Nature Portfolio wishes to improve the reproducibility of the work that we publish. This form provides structure for consistency and transparency in reporting. For further information on Nature Portfolio policies, see our Editorial Policies and the Editorial Policy Checklist.

Statistics

For all statistical analyses, confirm that the following items are present in the figure legend, table legend, main text, or Methods section.

n/a	Confirmed	
☐	☒	The exact sample size (*n*) for each experimental group/condition, given as a discrete number and unit of measurement
☐	☒	A statement on whether measurements were taken from distinct samples or whether the same sample was measured repeatedly
☐	☒	The statistical test(s) used AND whether they are one- or two-sided<br>*Only common tests should be described solely by name; describe more complex techniques in the Methods section.*
☐	☒	A description of all covariates tested
☐	☒	A description of any assumptions or corrections, such as tests of normality and adjustment for multiple comparisons
☐	☒	A full description of the statistical parameters including central tendency (e.g. means) or other basic estimates (e.g. regression coefficient) AND variation (e.g. standard deviation) or associated estimates of uncertainty (e.g. confidence intervals)
☐	☒	For null hypothesis testing, the test statistic (e.g. F, t, r) with confidence intervals, effect sizes, degrees of freedom and P value noted<br>*Give P values as exact values whenever suitable.*
☒	☐	For Bayesian analysis, information on the choice of priors and Markov chain Monte Carlo settings
☒	☐	For hierarchical and complex designs, identification of the appropriate level for tests and full reporting of outcomes
☐	☒	Estimates of effect sizes (e.g. Cohen's d, Pearson's r), indicating how they were calculated

Our web collection on statistics for biologists contains articles on many of the points above.

Software and code

Policy information about availability of computer code

Data collection	No software was used
Data analysis	The R codes used to analyse the data in this study have been described in the article

For manuscripts utilizing custom algorithms or software that are central to the research but not yet described in published literature, software must be made available to editors and reviewers. We strongly encourage code deposition in a community repository (e.g. GitHub). See the Nature Portfolio guidelines for submitting code & software for further information.

Data

Policy information about availability of data

All manuscripts must include a data availability statement. This statement should provide the following information, where applicable:

- Accession codes, unique identifiers, or web links for publicly available datasets
- A description of any restrictions on data availability
- For clinical datasets or third party data, please ensure that the statement adheres to our policy

The datasets generated during and/or analysed during the current study are available from the corresponding author on reasonable request.

Field-specific reporting

Please select the one below that is the best fit for your research. If you are not sure, read the appropriate sections before making your selection.

☐ Life sciences ☐ Behavioural & social sciences ☒ Ecological, evolutionary & environmental sciences

For a reference copy of the document with all sections, see nature.com/documents/nr-reporting-summary-flat.pdf

Ecological, evolutionary & environmental sciences study design

All studies must disclose on these points even when the disclosure is negative.

Study description	A 250-day microcosm experiment with 15N-labeled crop residues incorporated into a typical Mollisol was performed to reveal how microbial metabolic processes affect soil organic carbon accumulation in responses to differences in N supply from residues. There were hree treatments, i.e. 1) maize residue amendment, 2) soybean residue amendment and 3) no-residue control in a randomized complete block design. There were 15 replicates per treatment for five sampling dates. At sampling date, three replicates from each treatment were sampled.
Research sample	This study was conducted in a Mollisol, which is the most typical fertile farming soil distributed across the world. There were 15 replicates per treatment for five sampling dates. At 7, 30, 60, 100 and 250 days of incubation, three replicates from each treatment were randomly sampled. With MiSeq and HiSeq sequencing, soil bacterial and fungal community phylogenomic data and metagenomic data were generated.
Sampling strategy	Soil was collected from a typical corn-soybean rotation farming system, which is representative in Mollisol region. In the incubation experiment, we had 5 sampling times and on each time point, we undertook three replicates for further analyses. As the microcosm experiment ensure small variation between replicates. Three replicates are reasonable for generating reliable data sets.
Data collection	Data collection was carefully undertaken according to standard experimental procedures. The authors, Xie Z, Yu Z and Li Y collected data together and cross checked data to ensure the data quality. Some repeat measurements were conducted by other authors to confirm the consistency of data generation.
Timing and spatial scale	As the crop residue amendment influences microbial community and soil chemical properties intensively at the initial period. The sampling gap was small, and enlarged over time. Thus, sampling dates were 7, 30, 60, 100 and 250 days of incubation for DNA sequencing, microbial biomass, soil organic carbon analyses. Due to similar reason above, the microbial respiration was measured every three days in the first two weeks, weekly from day 19 to day 117, every 10 days from day 118 to day 168, and every two weeks each time thereafter.
Data exclusions	No data were excluded from the analyses
Reproducibility	Regarding the microbial properties, we measured microbial biomass carbon and respiration twice and obtained very similar results between the two round measurements, demonstrating the reproducibility of relevant findings. A preliminary experiment with similar crop residues (soybean) amended into Mollisols was also performed and we successfully fund that microbial activity and soil organic carbon had similar response with this experiment, indicating the reproducibility of the experiment.
Randomization	Samples were randomly arranged and sampled. At each sampling time, three replicates were randomly collected.
Blinding	We coded the treatment labels during data acquisition, in which the treatments were not recognizable to the person who undertook measurements. Experimental biases are eliminated.

Did the study involve field work? ☐ Yes ☒ No

Reporting for specific materials, systems and methods

We require information from authors about some types of materials, experimental systems and methods used in many studies. Here, indicate whether each material, system or method listed is relevant to your study. If you are not sure if a list item applies to your research, read the appropriate section before selecting a response.

Materials & experimental systems

n/a	Involved in the study	
☒	☐	Antibodies
☒	☐	Eukaryotic cell lines
☒	☐	Palaeontology and archaeology
☒	☐	Animals and other organisms
☒	☐	Human research participants
☒	☐	Clinical data
☒	☐	Dual use research of concern

Methods

n/a	Involved in the study	
☒	☐	ChIP-seq
☒	☐	Flow cytometry
☒	☐	MRI-based neuroimaging
